# BOWiki: an ontology-based wiki for annotation of data and integration of knowledge in biology

**DOI:** 10.1186/1471-2105-10-S5-S5

**Published:** 2009-05-06

**Authors:** Robert Hoehndorf, Joshua Bacher, Michael Backhaus, Sergio E Gregorio, Frank Loebe, Kay Prüfer, Alexandr Uciteli, Johann Visagie, Heinrich Herre, Janet Kelso

**Affiliations:** 1 Department of Computer Science, Faculty of Mathematics and Computer Science, University of Leipzig, Johannisgasse 26, 04103 Leipzig, Germany; 2 Institute for Logics and Philosophy of Science, Faculty of Social Science and Philosophy, University of Leipzig, Beethovenstrasse 15, 04107 Leipzig, Germany; 3 Department of Evolutionary Genetics, Max Planck Institute for Evolutionary Anthropology, Deutscher Platz 6, 04103 Leipzig, Germany; 4 Research Group Ontologies in Medicine (Onto-Med), Institute of Medical Informatics, Statistics and Epidemiology (IMISE), University of Leipzig, Härtelstrasse 16-18, 04107 Leipzig, Germany; 5 Communications and Publications Services, International Rice Research Institute, College, 4030 Los Baños, Laguna, Philippines; 6 Auckland Bioengineering Institute, University of Auckland, Level 6, 70 Symonds St., Auckland, New Zealand

## Abstract

**Motivation:**

Ontology development and the annotation of biological data using ontologies are time-consuming exercises that currently require input from expert curators. Open, collaborative platforms for biological data annotation enable the wider scientific community to become involved in developing and maintaining such resources. However, this openness raises concerns regarding the quality and correctness of the information added to these knowledge bases. The combination of a collaborative web-based platform with logic-based approaches and Semantic Web technology can be used to address some of these challenges and concerns.

**Results:**

We have developed the BOWiki, a web-based system that includes a biological core ontology. The core ontology provides background knowledge about biological types and relations. Against this background, an automated reasoner assesses the consistency of new information added to the knowledge base. The system provides a platform for research communities to integrate information and annotate data collaboratively.

**Availability:**

The BOWiki and supplementary material is available at . The source code is available under the GNU GPL from .

## Introduction

Biological ontologies have been developed for a number of domains, including cell structure, organisms, biological sequences, biological processes, functions and relationships. These ontologies are increasingly being applied to annotate and classify biological data. Data annotations with ontological categories provide an explicit description of specific features of the data, which are intended to enable users to integrate, query and reuse the data in ways previously not possible, thereby significantly increasing the data's value [1].

Developing and maintaining these ontologies requires manual creation, deletion and correction of concepts and their definitions within the ontology. Additionally, the annotated biological data must be maintained and new annotations created. In order to overcome the growing knowledge acquisition bottleneck, several authors suggest using community-based tools such as wikis for the description, discussion and annotation of the functions of genes and gene products [2-4]. To provide a useful resource for the scientific community, such a wiki must permit the acquisition of structured knowledge in addition to capturing knowledge in the form of free text.

However, an open approach like wikis frequently raises concerns regarding the quality of the information captured. The information represented in the wiki should adhere to particular quality criteria, such as internal consistency (the wiki content does not contain contradictory information) and consistency with biological background knowledge (the wiki content should be factually accurate). Logic-based tools can be employed to address some of these concerns. We have developed the BOWiki, a wiki system that uses a core ontology together with an automated reasoner to maintain a consistent knowledge base. It is specifically targeted at small- to medium-sized communities.

## Motivation

To facilitate the quick collaborative acquisition of knowledge, wiki systems can be used [5]. They permit the rapid creation and maintenance of knowledge in the form of free text. To enable the reuse of the captured knowledge for additional scientific analyses and queries, *semantic wikis *add a formal knowledge representation layer on top of the text-centered wiki functionality [6].

With collaborative knowledge acquisiton, maintaining the knowledge base's quality is of particular importance.

Several sources of errors can be identified. First, an entry in a knowledge base may not correspond to reality. For example, a statement to the effect that all African elephants have purple skin color is factually incorrect. This error can be detected by humans who review the information about African elephants and know that the fact in the knowledge base is incorrect. Automatic detection is more difficult. Formal theories about the domain of discourse must be available for automatic detection of incorrect knowledge to work. In particular, a formal theory that contains a statement about all African elephants having gray skin color and that states gray and purple are distinct colors can be used to detect a contradiction between the asserted statement and the theory.

Automated detection of this kind requires that large parts of biological knowledge are formalized and represented in a form that can be used for automated inferences, a state of affairs which is far from being achieved. We believe that completely automated detection of factually inaccurate knowledge is unfeasible. Alternatively, manual detection can be supported by providing easy access to the inferences drawn from asserted knowledge. Someone who is unaware of an African elephant's properties, but knows that all elephants are gray can identify as incorrect the assertion that African elephants are purple more easily when additional inferences are presented.

In contrast to factual correctness, internal consistency is easier to maintain through automated means. A set of statements is inconsistent if it contains a contradiction. Automated reasoners [7] can detect inconsistencies for a number of formalisms, including the computable fragment of the Web Ontology Language (OWL) [8]. To detect an inconsistency through reasoning, the representation formalism must offer to express negation, either explicitly or implicitly, in the knowledge base. OWL exhibits both types, e.g. the use of negated classes (explicit) and disjointness statements (implicit negation).

Another error source is the use of conflicting ontologies by the agents that collaborate in the knowledge base construction. Terms in ontologies may refer to different concepts, and therefore to reality in different ways. Many examples can be found in cases where the same term names concepts assuming an implicit context. For instance, there are at least five distinct concepts referred to as *cell wall *in various biological sub-domains. All those concepts are defined differently and may be correct in appropriate contexts, but none is *a priori *preferable. Formal ontologies can be used in order to make such distinctions explicit and thus to fix the intended meaning of a vocabulary to some extent. Therefore, they provide a means to support a common understanding of a basic vocabulary. Automated reasoners can then be used together with formalized ontologies to verify the consistency of an assertion in the knowledge base with respect to an accepted background ontology.

While most unwanted statements in a knowledge base are false, even true statements may reduce the quality of a knowledge base. Knowledge is widely considered to be justified true belief [9]. A belief, independent of its truth or falsehood, that lacks *justification *should not be included in a knowledge base. The justification of a belief in the knowledge base cannot be identified automatically, as the statement is often contained in a publication, webpage, or other source. The association of a statement with its source helps users to evaluate whether the statement should remain included in the knowledge base.

Summarizing, a collaboratively maintained knowledge base should provide easy access to inferences drawn from asserted knowledge to simplify the detection of incorrect assertions, maintain internal consistency, enforce the use of a common background ontology and permit the inclusion of justifications for assertions. These principles form the foundation of the quality control mechanisms that are implemented in the BOWiki software.

## System description

The BOWiki is a semantic wiki based on the MediaWiki software [10]. In addition to the text-centered collaborative environment common to wikis, a semantic wiki provides the user with an interface for entering structured data [11]. This structured data can subsequently be used to query the data collection.

**Figure 1:**
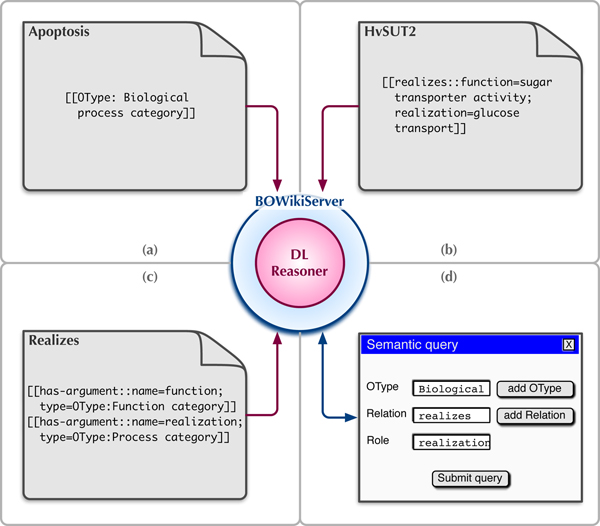
**Overview of basic BOWiki functionality**. (a) The OType statement is used to declare the entity described by a wikipage to be an instance of a certain type. The syntax for using a defined relation is shown in (b). Note that HvSUT2 implicitly fills a third argument role of realizes, since the relation is used on this page. For every relation, this implicit role is called the subject role. The relation **realizes **is therefore a ternary relation. (c) A relation's arguments are defined using the has argument statement. The example shows the definition for two roles and their restriction to specific OWL categories. All data stored in the BOWiki can be queried for, e.g. using the query form illustrated in part (d).

The BOWiki extends the MediaWiki's capabilities. It allows users to characterize the entities specified by wikipages as *instances *of ontological categories, to *define new relations *within the wiki, to *interrelate *wikipages and to *query *for wikipages satisfying selected criteria [12]. In particular, the BOWiki adds the following features to the MediaWiki software (for details see figure 1 and table 1): typing of wikipages (table 1), *n*-ary semantic relations among wikipages (table 1), semantic search and retrieval, reasoner support for content verification, adaptability to an application domain, import of bio-ontologies for local accessibility and simple reuse, graphical ontology browsing and the export of the wiki content into OWL [8].

**Table 1:** Syntax and semantics of the BOWiki extensions.

**BOWiki syntax**	**OWL abstract syntax**
*Generic*	
1 [[OType:C]]	Individual(**page **type(C))
2 [[R::page2]]	Individual(**page **value(R page2))
3 [[R::role1 = page1;...;roleN = pageN]]	Individual(R-id type(R))
	Individual(R-id value(subject **page**))
	Individual(R-id value(R-role1 page1))
	⋮
	Individual(R-id value(R-roleN pageN))
4 [[has-argument:: name = roleName;type = OType:C]]	SubClassOf(**page **gfo:Relator)
	ObjectProperty(R-roleName domain(**page**) range(C))

*Examples*	
1 on page Apoptosis: [[OType:Category]]	Individual(Apoptosis, type(Category))
2 on page Apoptosis: [[CC-isa::Biological_process]]	Individual(Apoptosis value(CC-isa Biological_process))
3 on page HvSUT2: [[Realizes:: function = Sugar_transporter_activity; process = Glucose_transport]]	Individual(Realizes-0 type(Realizes))
	Individual(Realizes-0 value(Realizes-subject HvSUT2))
	Individual(Realizes-0 value(Realizes-process Glucose_transport))
4 on page Realizes: [[has-argument:: name = function; type = OType:Function_category]]	SubClassOf(Realizes gfo:Relator))
	ObjectProperty(Realizes-function domain(Realizes) range(Function_category))

The table shows the syntax constructs used in the BOWiki for semantic markup. The second column provides a translation into OWL, following [23]. (**page **refers to the wikipage in which the statement appears; "R-id" is a name for an individual whose "id" part is unique and generated automatically for the occurrence of the statement). Because OWL has a model-theoretic semantics, this translation yields a formal semantics for the BOWiki syntax. In the lower half of the table we illustrate each construct with an example and present its particular translation to OWL.

We consider both adaptability to the application domain and content verification as the BOWiki's two outstanding novel features. Adaptability means that during setup, the software reads an OWL ontology selected by the user that provides a type system for the wikipages and the relations that are available to connect them. New relations can be introduced using specific wiki syntax, while the types remain fixed after setup.

While semantic wikis allow for the structured representation of information, they often provide little or no quality control and do not verify the consistency of captured knowledge. Using the imported ontology as a type system in the BOWiki provides additional background knowledge about the selected domain. This background knowledge is used to check user-entered, semantic content by means of an OWL reasoner. Currently, the performance of automated reasoners remains a limiting factor. Nevertheless, the reasoner delivers a form of quality control for the BOWiki content that should be adopted wherever feasible.

The BOWiki was primarily designed to describe biological data using ontologies. In conjunction with a core ontology [13] for biology like GFO-Bio [14] or BioTop [15], the BOWiki can be used for this purpose. A biological core ontology provides very general categories of the biological domain. More specific categories of biological sub-domains can be drawn from ontologies in the Open Biomedical Ontologies (OBO) [16]. In this connection, we developed a module that allows OBO flatfiles [17], the established data format for OBO ontologies, to be imported into the BOWiki. By default, these ontologies are only accessible for reading; they are neither editable nor considered in the BOWiki's reasoning. Users can then create wikipages containing information about biological entities, and describe the entities both in natural language text and in a formally structured way. For the latter, they can relate the described entities to categories from the OBO ontologies, and these categories are then made available for use by the BOWiki reasoning.

In contrast to annotating data with ontological categories, i.e., asserting an undefined association relation between a biological datum and an ontological category, it is possible in the BOWiki to define precisely the relation between a biological entity (e.g. a class of proteins) and another category: a protein may not only be *annotated *to *transcription factor activity*, *nucleus*, *sugar transport *and *glucose*. In the BOWiki, it may stand in the *has_function *relation to transcription factor activity; it can be *located_at *a nucleus; it can *participate_in *a *sugar transport *process; it can *bind *glucose. This use of distinct relations for linking data to categories allows for refined querying of the wiki contents. It is possible to define new relations in the BOWiki. These relations can be *n*-ary relations, i.e., they can have more than two arguments. Relations are defined on a special wiki page by specifying the relation's name, the names of their argument slots (relational roles [18]) and the types of the entities that can fill the argument slots.

The BOWiki can be used to describe not only data, but also biological categories, or to create relations between biological categories. As such, the BOWiki could further be used to create so-called cross-products [16,19] between different ontologies.

## Implementation

Within our MediaWiki extension, users can specify the type of entity described by a wikipage (see table 1). One of the central ideas of the BOWiki is to provide a pre-defined set of types and relations (and corresponding restrictions among them). We deliver the BOWiki with the biological core ontology GFO-Bio [14], but other foundational ontologies may be used, e.g. BioTop [15], BFO [20] and DOLCE [21]. Technically, any *consistent *OWL-DL [8] file can be imported as the type system. Types are modeled as OWL classes and binary relations as OWL properties. Relations of higher arity are modeled according to use case 3 in [22], i.e., as classes whose individuals model relation instances. Ontological justification for using this pattern can be found in [18,23]. Wikipages as descriptions of instances of types give rise to OWL individuals, which may be members of OWL classes.

An OWL ontology can provide background knowledge about a domain in the form of axioms that restrict the basic types and relations within the domain. This allows for automatic verification of parts of the semantic content created in the BOWiki: users may introduce a new page in the wiki and describe some entity; they may then add type information about the described entity; this added type information is then automatically verified. The verification checks the logical consistency of the BOWiki's content – as OWL individuals and properties relating them – with the restrictions of the ontology's types and relations, like those in GFO-Bio. Therefore, OWL reasoning enforces the commitment to a common conceptualization of a domain, as far as it is formalized in the background ontology.

**Figure 2:**
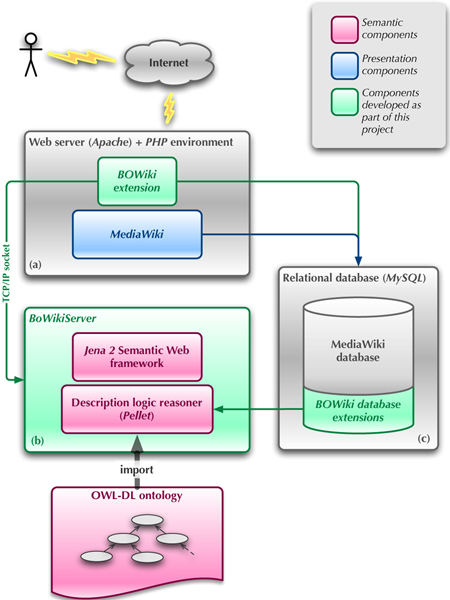
**BOWiki architecture**. (a) The BOWiki extension to the MediaWiki software processes the semantic data added to wiki pages. The semantic data is subsequently transferred to the BOWikiServer using a TCP/IP connection. (b) To evaluate newly entered data or semantic queries, the BOWikiServer requires an ontology in OWL-DL format (provided during installation of the BOWiki). Consistent semantic data will be stored. If an inconsistency is detected, the edited page is rejected with an explanation of the inconsistency. The BOWikiServer currently uses the Jena 2 Semantic Web Framework together with the Pellet reasoner. (c) After successful verification the semantic data is stored in a separate part of the SQL database.

The BOWiki uses a description logic [24] reasoner to perform those consistency checks. A layer of abstraction is needed between the BOWiki application and the description logic reasoner in order to support more than one type of reasoner. While the DIG protocol [25] provides such an abstraction layer and is implemented by many description logic reasoners, it does not support operations required by the BOWiki. Among the missing features are the removal of instances, rollbacks of the knowledge base or explanations of detected inconsistencies. To address these problems, we implemented the BOWikiServer, a stand-alone server that provides access to a description logic reasoner using the Jena 2 Semantic Web Framework [26] and a custom-developed protocol. A schema of the BOWiki's architecture is illustrated in figure 2.

Whenever a user edits a wikipage in the BOWiki, the consistency of the changes with respect to the core ontology is verified using the BOWikiServer. Only consistent changes are permitted. In the event of an inconsistency, an explanation for the inconsistency is given, and no change is made until the user resolves the inconsistency. The inconsistency can be resolved through the modification of the new, conflicting statement, or modifications of statements that are already contained in the knowledge base.

In addition to verifying the consistency of newly added knowledge, the BOWikiServer can perform complex queries over the data contained within the wiki. Queries are performed as retrieval operations for description logic concepts [24], i.e., as queries for all individuals that satisfy a description logic concept description.

The performance of the description logic reasoner employed in the BOWiki limits the performance of the overall system. A comprehensive empirical study of a BOWiki installation populated with real-world data is subject to future work. Automated tests appear unfeasible due to a large number of indeterminate parameters of such data. However, evaluations of the Pellet reasoner on real-world data sets in other domains [27] may provide an estimate of BOWiki's performance.

## Discussion

### Using different reasoners

The BOWikiServer provides a layer of abstraction between the description logic reasoner and the BOWiki.

Depending on the description logic reasoner used, different features can be supported. Currently, the BOWikiServer uses the Pellet reasoner [7]. Pellet supports the explanation of inconsistencies, which can be shown to users to help them in correcting inconsistent statements submitted to the BOWiki. It also supports the nonmonotonic description logic ALCK with the auto-epistemic **K **operator [28]. This permits both open- and closed-world reasoning [29] to be combined. Several practical applications of this have been discussed for the integration of ontologies in biology [14] and in the context of the Semantic Web [30], e.g. "epistemic querying" as enhanced querying capability of a system. On the other hand, reasoning in the OWL description logic fragment is highly complex [31]. It is possible to use reasoners for weaker logics to overcome the performance limitations encountered with Pellet.

### Comparison with other approaches

WikiProteins [4] is a software project based also on the MediaWiki software, focused on annotating Swissprot [32]. Similar to the BOWiki, it utilizes ontologies like the Gene Ontology [1] and the Unified Medical Language System [33] as a foundation for the annotation. It is generally more targeted at creating and collecting definitions for terms than on capturing knowledge in a logic-based and ontologically founded framework. As a result, it contains a mashup of lexical, terminological and ontological information. In addition, WikiProteins neither supports *n*-ary relations nor provides a description logic reasoner to retrieve or verify information. It therefore lacks the quality control and retrieval features that are central to the BOWiki. On the other hand, because of the different use-cases that WikiProteins supports, it is designed to handle much larger quantities of data than the BOWiki, and it is better suited for creating and managing terminological data.

The Semantic Mediawiki [11] is another semantic wiki based on the Mediawiki software. It is designed to be applicable within the online encyclopedia Wikipedia. Because of the large number of Wikipedia users, performance and scalability requirements are much more important for the Semantic Mediawiki than for the BOWiki. Therefore, it also provides neither a description logic reasoner nor ontologies for content verification.

The IkeWiki [34], like the BOWiki, includes the Pellet description logic reasoner for classification and verification of consistency. However, parts of the IkeWiki's functionality require users to be experts in either Semantic Web technology or knowledge engineering. As a consequence, the BOWiki lacks some of the functionality that the IkeWiki provides (such as creating and modifying OWL classes) as it targets biologist users, most of whom are not trained in knowledge engineering. On the other hand, the IkeWiki lacks some functionality included in the BOWiki, most notably the ability to process ontologies in the OBO Flatfile Format.

## Conclusion

We developed the BOWiki as a semantic wiki specifically designed to capture knowledge within the biological and medical domains. It has several features that distinguish it from other semantic wikis and from similarly targeted projects in biomedicine, most notably its ability to verify its semantic content for consistency with respect to background knowledge and its ability to access external OBO ontologies.

The BOWiki is intended to enable a scientific community to annotate biological data rapidly. This annotation can be performed using biomedical ontologies. In addition to data annotation, the specific type of relations between entities can be made explicit. It is also possible to integrate different biological knowledge bases by creating partial definitions for the relations and categories used in the knowledge bases.

The BOWiki employs a type system to verify the consistency of the knowledge represented in the wiki. The type system is provided in the form of an OWL ontology. If the type system is a core ontology for a domain (i.e., it provides background knowledge and restrictions about the categories and relations for the domain), its use contributes to maintaining the ontological adequacy of the BOWiki's content, and thereby the content's quality.

## Competing interests

The authors declare that they have no competing interests.

## Authors' contributions

KP and RH conceived the initial idea, JB, MB, RH and AU implemented the BOWiki and the BOWikiServer. HH, RH and FL provided logical and ontological support. JB, MB, SG, HH, RH, JK, FL, AU and JV participated in the design of the BOWiki software. JK supervised the project. HH, RH, JK and FL wrote this paper. All authors participated in discussion and revision of the paper. All authors read and approved the final manuscript.
